# Supplementary material for: Study protocol to establish a prospective cohort for the study of phenotypic clusters, progression pathways, and outcomes of frailty and dependence: The CohorFES
Source: PLoS One. 2026 Mar 26;21(3):e0345101. doi: 10.1371/journal.pone.0345101 (PMC13020820; doi:10.1371/journal.pone.0345101)
Supplement: S1 File — (PDF) [file pone.0345101.s001.pdf]

GOBIERNO DE ESPAÑA | MINISTERIO DE CIENCIA, INNOVACIÓN Y UNIVERSIDADES | Instituto de Salud Carlos III

Subdirección General de Evaluación y Fomento de la Investigación

Unión Europea
Fondo Europeo de Desarrollo Regional
"Una manera de hacer Europa"

2019

HEALTH RESEARCH PROJECTS PROPOSAL APPLICATION FORM

Application No

PRINCIPAL INVESTIGATOR (PI) DETAILS

SURNAME NOGUES SOLAN **NAME** FRANCESC XAVIER

DNI/NIE: 37318757S **BIRTH DATE (DD/MM/YYYY):** 14/09/1959 **GENDER (M/F):** M

PHONE: 934265499 **ORCID:** 0000-0002-5537-1859

ACADEMIC BACKGROUND OF THE PRINCIPAL INVESTIGATOR

DEGREE	CENTRE	DATE
MEDICINE	UNIVERSITAT AUTONOMA DE BARCELONA	30/06/1982
		23/2/2015

PhD	CENTRE	DATE
MEDICINE	UNIVERSITAT AUTONOMA DE BARCELONA	24/02/1992
AGEING AND FRAILTY	UNIV. NACIONAL DE EDUCACION A DISTANCIA (UNED)	23/2/2015

EMPLOYMENT BACGROUND OF THE PRINCIPAL INVESTIGATOR

CURRENT POSITION
HEAD OF INTERNAL MEDICINE DEPARTMENT

STARTING DATE
21/12/2015

INSTITUTION PARC DE SALUT MAR

CENTRE/FACULTY/COLLEGE/INSTITUTE:
INSTITUT HOSPITAL DEL MAR D INVESTIGACIONS MEDIQUES

DEPT./UNIT/SECTION: MUSCULOSKELETAL RESEARCH

EMAIL/S xnogues@parcdesalutmar.cat

AFFILIATION

- ○ CIVIL SERVANT
- ○ STATUTORY
- ◉ PERMANENT CONTRACT
- ○ TEMPORARY CONTRACT
- ○ PROFESSOR EMERITUS

PROFESSIONAL OR SCIENTIFIC BACKGROUND

STARTING/END YEARS	POSITION	INSTITUTION
2007-2015	HEAD OF CLINIC INTERNAL MEDICINE	PARC DE SALUT MAR
1991-2007	CONSULTANT OF INTERNAL MEDICINE	PARC DE SALUT MAR
1985-1990	RESIDENT MIR	HOSPITAL ESPERANÇA
1996-	ASSOCIATE PROFESSOR	UNIV. AUTONOMA BARCELONA

HEALTH RESEARCH PROJECTS
PROPOSAL APPLICATION FORM

Application No.:

DETAILS OF THE CO-PRINCIPAL INVESTIGATOR (CO-PI)

SURNAME NAME

DNI/NIE: BIRTH DATE (DD/MM/YYYY): GENDER (M/F):

PHONE: ORCID:

ACADEMIC BACKGROUND OF THE CO-PRINCIPAL INVESTIGATOR (CO-PI)

DEGREE	CENTRE	DATE
PhD	CENTRE	DATE

EMPLOYMENT STATUS OF THE CO-PRINCIPAL INVESTIGATOR (CO-PI)

CURRENT POSITION STARTING DATE

INSTITUTION

CENTRE/FACULTY/COLLEGE/INSTITUTE

DEPT./UNIT/SECTION:

EMAIL/S

AFFILIATION
- ◯ CIVIL SERVANT
- ◯ STATUTORY
- ◯ PERMANENT CONTRACT
- ◯ TEMPORARY CONTRACT
- ◯ PROFESSOR EMERITUS

PROFESSIONAL OR SCIENTIFIC BACKGROUND

STARTING/END YEARS	POSITION	INSTITUTION

Application No.:

PRINCIPAL INVESTIGATOR:

HEALTH RESEARCH PROJECT APPLICATION FORM
TRAINING CAPACITY OF THE PI AND CO-PI

- Supervised dissertations within the last five years. Please state: PhD student, title, year, University/Institution
- Human resources obtained as team leader in national or international calls within the last 5 years. Please state: program (i.e. Ramon y Cajal, Miguel Servet, Rio Hortega, Marie Curie…), researcher, funding agency, duration (from… to…)

(Please only use the space provided below)

Superviser of PhD thesis: Roberto Güerri Fernandez
In vivo mocroidentation for the assessment of bone material properties Universitat Autònoma de Barcelona 04/07/2013
Superviser PhD thesis: Maria Rodriguez Sanz
Musculoskeletal side effects of aromatase inhibitors in women with breast cancer. Universitat Autònoma de Barcelona 15/12/2016
Superviser PhD thesis: Cristina Carbonell Abella
Femoral fracture Hospital management in Spain. Variability and tendencies. Universitat Autònoma de Barcelona 22/09/2016
Superviser PhD thesis Lidia García Gibert
Genetic influence on the evolution of bone mass after parthyroidectomy in patients with hyperparathyroidism. Universitat Autónoma de Barcelona 13/07/2016
Superviser PhD thesis: Daniel Martinez Laguna
Effect of Diabetes type 2 on the Osteoporotic Fracture incidence: DIAFOS study. Universitat Autònoma de Barcelona 26/09/2017
Superviser PhD thesis: Verónica Medialdea Dziatzko
Analysis of short and long effects of a thermal care in patients with chronic diseases. Universitat Autònoma de Barcelona 30/11/2018
Superviser PhD thesis: Farid Taymouri
Reference Data for bone mineral strength (BMSi) measured by impact microindentation. Universitat Autnoma de Barcelona 8/2/2019

AGÈNCIA DE GESTIÓ AJUTS UNIVERSITARIS DE RECERCA 2009 SGR 818 budget: 50.056 euros

LEADERSHIP IN INTERNATIONAL R&D&I PROJECTS OF THE PI AND THE CO-PI

Project: GENOMOS European Commission QURT-2001-02629
117.924 euros

Study to Determine the Efficacy and Safety of Romosozumab in the Treatment of Postmenopausal Women With Osteoporosis (ARCH), international AMG 785
Amgen 13.198

Effect of Teriparatide on Hip Fracture Healing
Entitat finançadora: Lilly SA 5.325

Study Evaluating Changes In Bone Mineral Density (BMD), And Safety Of Rhbmp-2/CPM In Subjects With Decreased BMD
WYETH FARMA S.A 64.200 euros

Subdirección General de Evaluación y Fomento de la Investigación

Unión Europea
Fondo Europeo de Desarrollo Regional
"Una manera de hacer Europa"

2019

Application No.:

TITLE: **Analysis of phenotypic clusters, progression paths and outcomes of frailty and dependence: The Spanish CohorFES Study and the SIDIAP database.**

PRINCIPAL INVESTIGATOR: FRANCESC XAVIER NOGUES SOLAN

CO-PRINCIPAL INVESTIGATOR:

TYPE OF PROJECT (●) **INDIVIDUAL** ( ) **COORDINATED** ( ) **MULTICENTER**

NAME OF THE COORDINATING PI:
(Only in coordinated projects)

DURATION: ☒ **3 YEARS**

ABSTRACT (Objectives and Methodology of the Project) **(Please only use the space provided below)**

Frailty has become a major problem for the health system, but also a window of opportunity to fight against disability through preventive strategies focused on the detection and treatment of frailty in all settings. However, no systematic strategies of screening and early detection are available in clinical settings. This project aims to look for clinical and biological phenotypic clusters that drive through the different stages of frailty and to identify the underlying mechanisms with the main aim of knowing the trajectories leading to disability and the potential for treatment. Moreover, validation of Frailty Trait Scale 5 (FTS5) will be performed as a easy model to be implemented in Primary care and Hospital scope. Two population-based cohorts will be used for frailty phenotyping: A new stablished Spanish clinical cohort (CohorFES) into the CIBERFES and the SIDIAP. Using data-driven artificial intelligence methods, cluster partition models (k-means and hierarchical clustering) will group together individuals with similar characteristics defining phenotypes of frailty/ pre-frailty. Then, by pre-established criteria, the proposed clustering solution will be evaluated and labelled according to their defining characteristics. Further, latent class analysis models (mixed growth models) will be applied for the identification of profiles of longitudinal trajectories. Final outcomes will be health care resources use, hospital admissions and mortality. Survival models (Kaplan-Meier and Cox proportional hazard models or competing risk Fine-Gray regression) will analyse the impact of the identified cross sectional and longitudinal frailty phenotypes on these final outcomes. Rapid progression and deterioration in several clusters of frailty will be explored in order to identify the biological markers involved in the frailty progress for an early identification and monitoring of this rapid deterioration.

TÍTULO: Análisis de agrupaciones fenotípicas, trayectorias de progresión y factores de fragilidad y dependencia: estudio de una cohorte española CohorFES y la base de datos SIDIAP.

RESUMEN (Objetivos y Metodología del proyecto) **(Ajústese al espacio disponible)**

La fragilidad se ha convertido en un problema importante para el sistema de salud, pero también en una ventana de oportunidad para combatir la discapacidad a traves de un abordaje preventivo centrado en la detección y tratamiento de la fragilidad. Sin embargo no existen estrategias sistemáticas para su detección precoz y tratamiento en los medios clínicos. El objetivo de este proyecto es buscar clústeres fenotípicos clínicos y biológicos a través de las diferentes etapas de la fragilidad e identificar los mecanismos subyacentes con el objetivo de conocer las trayectorias que conducen a la discapacidad y su potencial de tratamiento. Además, se pretende validar la Escala de Rasgos de Fragilidad 5 (FTS5) como un modelo fácil de implementar en el ámbito de Atención primaria y en el Hospital. Se utilizarán dos cohortes basadas en la población para el fenotipado de fragilidad: una nueva cohorte clínica española (CohorFES) dentro del CIBERFES y la SIDIAP. Mediante el uso de métodos de inteligencia artificial, los modelos de partición de agrupación (k-medias y agrupación jerárquica) agruparán a los individuos con características similares que definan los fenotipos de fragilidad / prefragilidad. Luego, según criterios preestablecidos, los distintos grupos se evaluarán y etiquetarán de acuerdo con sus características definitorias. Además, se aplicarán modelos de análisis de clases latentes (modelos de crecimiento mixto) para la identificación de perfiles de trayectorias longitudinales desde la robustez a la discapacidad. Los resultados abarcan el uso de los recursos de atención de salud, los ingresos hospitalarios y la mortalidad. Los modelos de supervivencia (modelos de riesgo proporcional de Kaplan-Meier y Cox o regresión Fine-Gray de riesgo competidor) analizarán el impacto de los fenotipos de fragilidad transversal y longitudinal identificados en estos resultados finales. Se explorará la progresión rápida y el deterioro en varios grupos de fragilidad a fin de identificar los marcadores clínicos y biológicos implicados en el progreso de la fragilidad para una identificación precoz y el monitoreo de este rápido deterioro.

Application No.:
PRINCIPAL INVESTIGATOR:

HEALTH RESEARCH PROJECT APPLICATION FORM
BACKGROUND AND STATE-OF-THE-ART

Project's aim, background and state-of-the-art of the scientific and technical knowledge, and national or international groups working in the same specific or related lines.
Please state references in the next item: Relevant references **Max. 3 pages (15,700 characters)**

Frailty is one of the major challenges of the 21st Century, and a top priority for national and international organisms like the WHO or the European Parliament. This has put frailty as one of the top prioritie in the biomedical research agenda of the European Commission [1,2]. Frailty is constituted by a physiological background of increased vulnerability and impaired resilience to stressors (i.e. diseases, external agents, drugs tolerability and toxicity) due to the combined effect of the aging process and some chronic diseases which drives to a final stage of dependency and disability with a sharp impact in quality of life, health and social resources consumption, hospitalization and death (2).
It is well-known the relevance of frailty, its detection, and management since we are aware about their reversibility (3), the costs on the health systems (4), and its potential impact in clinical settings (5). This huge amount of new information has provided relevant clues to the best understanding of frailty in community-dwelling older people, including the tools for its assessment, the trajectories among the different frailty and disability statuses and the factors potentially implicated. However, in a clear contrast with this abundancy of data in non-clinical settings, there is a lack of strong data in the setting where the prevalence of frailty is the highest and where the risks for developing its most serious adverse consequences are the highest: the cinical settings (6), promoting the need of a clinical transition led by the right detection and management of frailty/prefrailty. There is hence an urgent need for a better screening and diagnosis of frailty, its trajectories and the determinants of these separate trajectories depeding upon both the characteristics of frailty in each patient (associated or not with sarcopenia, or cognitive impairment or different clusters of chronic diseases). But also for a better understanding of the mechanisms and pathways that lead to the development of frailty syndromes in the community and its different evolutions in order to make the easiest not only its detection, but also its prognosis depending upon the presence of several biomarkers (laboratory biomarkers, but also image biomarkers or clinical biomarkers) and its treatment, all of them in clinical settings, assessing both out- and in-older patients.
While the different categories of the syndrome based on the severity of the observed deficits (robust, frail, pre-frail) are quite well defined and characterised from an epidemiological point of view (7,8), there is a scarcity of data on the functional pathways between these diagnostic categories (and, among them, disability), and this is specially true in clinical cohorts. This is really shocking taking into account that one of the most relevant factors, if not the first one, associated with a poor evolution of frailty is to experience an episode of hospitalization (9).
This implies a loss of opportunity to investigate: 1) The clinical and biological factors profiling at-risk groups (phenotypic clusters) that drive the progression of the frailty syndrome from its early stages; 2) Potential interventions capable of preventing the progression to more advanced stages of frailty and disability, and 3) research into treatments that revert the problem to prior functional stages.
Recent developments in artificial intelligence using unsupervised machine learning algorithms allow the analysis of very large populations, and the identification of clusters of individuals that show different phenotypic characteristics [7] or that accumulate deficits [8] that define different types of frailty. Based on this information, longitudinal mixed growth models can enable the detection of groups that accumulate some of those phenotypic characteristics or deficiencies over time, clustering them and

studying their particularities in terms of associated factors, trajectories, biomarkers and responses to treatment (10,11). Our overarching goal is therefore to identify the critical subgroups of subjects at risk of progression from robustness to prefrailty and frailty and from there to their late stages, and the pathways that mediate this trajectory amongst community-dwelling Spanish subjects. It is worthy to mention that these trajectories although spontaneously tend to flow to an impairment of the functional status, can also spontaneously improve, although up to now there are no data about the factors associated with these different trajectories, mainly to those which are benefitial for the patients. In this same line, although we started to collect some information about the benefits of some treatments (that is, a benefit on functional trajectories through some interventions) previously mentioned, we do not have information about the phenotypic characteristics of the responders or non-responders, nor about the biomarkers associated with a good or poor response rate or prognosis. Accordingly, this research will unravel critical points and potentially vulnerable populations to whom further clinical and translational research should be targeted.

Other important issue in this field would be to find an easy tool to identify frailty and factors which could be implemented in our full outpatients list. In addition to the more classical instruments to assess frailty, several groups currently members of CIBERFES developed an instrument that overcome some of the problems raised by the more classical ones. The Frailty Trait Scale-FTS has shown a better predictive capacity for some outcomes in very old patients (12) living in the community. More recently, and as part of an EU-funded project (FRAILTOOLS) we have found that the full version of FTS is able to detect frailty in some clinical settings (Acute Care Geriatric Unit, Geriatric Service outpatient office and Primary Care), with a good predictive capacity for adverse outcomes (death, incident disability, deterioration in SPPB, falls and hospitalization) at 6-12-18 months (13). However the full version of FTS, composed by 14 items, takes around 15 minutes, making it unpractical in usual clinical conditions, where the time available by the physician or the nurse is lower. With this fact in mind two groups of CIBERFES involved in this research proposal (Complejo Hospitalario de Toledo and Hospital Universitario de Getafe) have developed a shorter version of only 5 items (the so called FTS 5). This shorter version takes as a mean 107 seconds, a time similar to the one needed to pass the FRAIL scale (89 seconds) and much shorter than the Frailty Phenotype (220 seconds) or the Frailty Index Questionaire-35 items (223 seconds). But more interestingly, FTS5 seems to offer a better classification of community-dwelling older people regarding the risks of death, hospitalization and incident disability at three years than the more classical tools Frailty Phenotype or Frailty Index (preliminary unpublished results). In addition, several studies showing trajectories of frailty in clinical settings suggest that the detection of changes in the trajectory depends critically on the characteristics of the tool used to detect and monitor frailty. In this regard, as previously stated, FTS5 offers promising results based upon the sensitivity to detect small changes shown by the full FTS. Hopefully, FTS5, which derivates from the FTS and seems to conserve its main characteristics in older adults living in the community, will have also this sensitivity to detect change, as it provides a score for each individual ranging from zero to fifty. Finally, the variables that compose the FTS5 (gait velocity, grip strength, BMI, PASE, and balance) can be incorporated to electronic instruments. This has been the case for the electronic frailty index (eFI), developed and validated in the British electronic records based on the Rockwood's frailty model (14) that would allow to assess the frailty profile after to consider 36 items or deficits at the same moment of visit by primary care o hospital physician or the more recent Hospital Frailty Risk Score based on clinical diagnoses that is able to predict death but showing only a fair concordance with the Frailty Phenotype and the Frailty Index (15).

The use of easy electronic tools has been useful not only in hospital care but also into routine primary care practice. Moreover, it would be easier to measure the adverse outcomes, including falls, delirium, disability, care home admission, hospitalization and mortality as it has been recently shown (16).

The findings of theoretical framework of frailty and pathways that drive to negative trajectories and worsening frailty status could be applied in the local and international guidelines. Recently eFI has been used in other countries and it has been demonstrated its utility to extract information of outcomes (16).

Although this type of research efforts can be afforded by single groups, the structure of CIBERFES offers several advantages making it a valuable intrument for succesfully carried out this kind of research work. It embraces several high-quality (all of them met the highly exigent conditions need to be qualified as a member of any CIBER and have been recently evaluated by international and national experts in the field) clinical groups that will work jointly with epidemiologists pertaining to these or some other groups inside the CIBER de Fragilidad y Envejecimiento Saludable-CIBERFES. Moreover, in the next years, the groups with expertise in basic science will provide the determinations to assess the role of different potential biomarkers. In addition to this newly stablished Spanish clinical cohort named CohorFES into the CIBERFES, other population-based cohort will be used, the SIDIAP cohort. SIDIAP database is a real-world (computerized medical records) big data that could provide eligible aged >75 patients. Its length of follow-up (up to 10 years of follow-up) readily available enables powerful analyses as the ones listed for one work package (WP) of the current project. The SIDIAP database contains deidentified information on socio demographics, lifestyle risk factors, clinical measurements, co-morbidity (recorded diagnoses using ICD-10 codes), prescriptions, and linked community pharmacy dispensations (as coded using the World Health Organization ATC catalogue). In addition, SIDIAP holds information on activities of daily living (Barthel) and cognitive function (Pfeiffer test), recorded during routine practice, for a substantial 354,457,and 346,576 people aged >75 years respectively. This, linked to hospital admissions data, constitutes a unique dataset for the study of frailty, its impact, and its possible treatment in non-institutionlized older people attending different clinical settings in hospital and Primary Care.

In conclusion, frailty is a highly relevant clinical conditions, able to predict in older people adverse outcomes even better than clinical diagnoses (17) and which offers oportunities for intervention dealying or avoiding its evolution toward disability. Inside this conceptual framework and taking into account the scarce data available in clinical settings about its diagnosis, trajectories and prognosis, the main goal of this project is to look for clinical and biological phenotypic clusters that drive through the different stages of frailty and to identify the underlying mechanisms that finally will trigger the disability, using an approach that has shown to be highly succesful in Spain (for instance, PREDIMED). The objective is the implementation of a systematic computerized routine in the electronic medical records for screening, early identification and monitoring of this rapid deterioration. In such a way, in addition to provide relevant data from this collaborative effort the current proposal also produced a strong research structure where other projects could be hanged thus multiplying its research outcomes.

Application No.:

PRINCIPAL INVESTIGATOR:

HEALTH RESEARCH PROJECT APPLICATION FORM
BACKGROUND AND STATE-OF-THE-ART. RELEVANT REFERENCES

Please list references of the quotes included in the previous item: background and state-of-the-art. (Max. 1 page)

1.-Morley JE, Cha B, Vellas B, Abellan van Kan G. Frailty Consensus: A Call to Action J Am Med Dir Assoc. 2013;14: 392 -7.
2.-Rodríguez-Mañas L, Féart C, Mann G, et al. Searching for an operational definition of frailty: a Delphi method based consensus statement: the frailty operative definition-consensus conference project. J Gerontol A Biol Sci Med Sci. 2013;68:62-7
3.- Trombetti A, Hars M, Hsu FC, et al. Effect of Physical Activity on Frailty: Secondary Analysis of a Randomized Controlled Trial. Ann Intern Med. 2018; 168: 309-316.
4.- Sirven N, Rapp T. The cost of frailty in France: Eur J Health Econ, 2016;
5.- Rodriguez-Mañas L, Fried LP. Frailty in the clinical scenario. Lancet. 2015; 385: e7-9.
6.- Rodriguez-Mañas L, Rodríguez-Artalejo F, Sinclair AJ. The Third Transition: The Clinical Evolution Oriented to the Contemporary Older Patient. J Am Med Dir Assoc. 2017; 18: 8-9.
7.- Fried LP, Tangen CM, Walston J, et al. Frailty in older adults: Evidence for a phenotype. J Gerontol A Biol Sci Med Sci 2001; 56: M146eM156.
8.- . Rockwood K, Mitnitski A. Frailty in relation to the accumulation of deficits. J Gerontol A Biol Sci Med Sci
9.- Gill TM, Gahbauer EA, Han L, Allore HG. The relationship between intervening hospitalizations and transitions between frailty states. J Gerontol A Biol Sci Med Sci 2011; 66A: 1238-43
10.- Strauss VY, Jones PW, Kadam UT, Jordan KP. Distinct trajectories of multimorbidity in primary care were identified using latent class growth analysis. J Clin Epidemiol. 2014 Oct;67(10):1163-71.
11.- Nderitu P, Doos L, Strauss VY, Lambie M, Davies SJ, Kadam UT. Analgesia dose prescribing and estimated glomerular filtration rate decline: a general practice database linkage cohort study. BMJ Open. 2014 Aug 19;4(8):e005581.
12.- García-García FJ, Carcaillon L, Fernandez-Tresguerres J, Alfaro A, Larrion JL, Castillo C, Rodriguez-Mañas L. A new operational definition of frailty: the Frailty Trait Scale.J Am Med Dir Assoc.2014;15:371.e7-371.e13.
13.- FRAILTOOLS: A comprehensive validation of tools to screen and diagnose frailty in different clinical and social settings to provide instruments for integrated care in older adults. Final Report. DG-SANTE, Brussels, 2019
14.- Clegg A, Bates C, Young J, et al. Development and validation of an electronic frailty index using routine primary care electronic health record data. Age Ageing. 2016;45:353-60.
15.- Gilbert T, Neuburger N, Kraindler J, et al. Development and validation of Hospital Frailty Risk Score focusing on older people in acute care settings using electronic hospital records: an observational study. Lancet 2018; 391: 1775-82
16.- Stow D, Matthews F, Barclay S, Iliffe S et al. Evaluating frailty scores to predict mortality in older adults using data from population based electronic health records: case control study. Age and Ageing 2018; 47: 564 -569.
17.- Castro-Rodríguez M, Carnicero JA, Garcia-Garcia FJ, Walter S, Morley JE, Rodríguez-Artalejo F, Sinclair AJ, Rodríguez-Mañas L. Frailty as a Major Factor in the Increased Risk of Death and Disability in Older People With Diabetes. J Am Med Dir Assoc. 2016; 17: 949-55.

Application No.:

PRINCIPAL INVESTIGATOR:

HEALTH RESEARCH PROJECT APPLICATION FORM
HYPOTHESIS AND OBJECTIVES

(Please only use the space provided below)

HYPOTHESIS

1.- Easy models of frailty evaluation is needed to implement the concept of frailty in the health outcomes

2.- The clustering of different clinical and biological phenotypes permits to identify differentiated longitudinal frailty pathways.

3.- The different phenotypes and clusters of frailty make diverse health outcomes: use of health services (both primary and secondary care), hospital admissions, and mortality.

4.- Rapid progression and deterioration in several clusters of frailty have to be explored in order to identify the biological markers involved in the frailty progress for an early identification and monitoring of this rapid deterioration.

OBJECTIVES

WP1. Easy models of frailty evaluation

1.- Creation of a prospective Spanish clinical cohort (CohorFES) into the CIBER de Fragilidad y Envejecimiento Saludable-CIBERFES at the Primary Care and Hospital.
2.- Validation of Frailty Trait Scale 5 (FTS) as a easy model to be implemented in Primary care and Hospital scope.
3.- To identify, through the electronic frailty index (eFI) and machine learning algorithms, frailty patients registered in SIDIAP on January 1st 2007, of age 65 or above that could be used in WP2 and WP3.

WP2. Identification of different phenotypes, clusters of frailty and trajectories

1.- To describe frailty phenotypes characterized by the clustering of different physiological deficiencies in the CohorFES and SIDIAP.
2. To identify differentiated longitudinal frailty pathways either by accumulation of deficiencies or by the characteristics of the accumulated deficiencies in the CohorFES and SIDIAP.
3. Explore, in a subgroup from a cluster of rapid progression, the clinical and biological markers that characterize early identification and monitoring of this rapid deterioration in the CohorFES.
4.- Creation of a CIBERFES Biobank where all the participants of CohortFES could invited to participated and blood and urine samples could be stored for future researches.

WP3. Frailty and health outcomes in CohorFES and SIDIAP

1. To analyze the existing relationship between the different phenotypes and the following health outcomes: use of health services (both primary and secondary care), hospital admissions, and mortality.

Application No.:

PRINCIPAL INVESTIGATOR:

HEALTH RESEARCH PROJECT APPLICATION FORM
COORDINATED PROJECTS

In the case of coordinated projects, COORDINATOR shall detail:
- **-Global objectives of the coordinated project, need of coordination and expected added value.**
- **-Specific objectives of each of the sub-projects (they shall also be included in each sub-project's application).**
- **-Interaction between different objectives, activities and sub-projects.**
- **-Coordination mechanisms for the effective implementation of the project.**

Max. 3 pages (15,700 characters)

Application No.:	
PRINCIPAL INVESTIGATOR:	

HEALTH RESEARCH PROJECT APPLICATION FORM
METHODOLOGY

Design, study subjects, variables, data collection and analysis, study limitations.

Max. 3 pages (15,700 characters)

Design:
A population-based cohort study including data of Primary and Secondary care (Hospitalization).

All procedures proposed in this project will be in accordance with the ethical standards of the institutional and/or national research committee and with the October, 2013 Helsinki Fortaleza Declaration, Brazil, or comparable ethical standards. The data confidentiality will be guaranteed in accordance with the Law Organic 3/2018, December 5, for the Protection of Personal Data and guarantee of digital rights and Regulation (EU) nº2016 / 679 of the European Parliament, and of the Council of April 27, 2016 of Data Protection (RGPD).

Participants:
WP1. 1 and 2. Patients recruited for the CohorFES
Women and men 65 years old or above visited in the outpatient clinics of participants centers; Primary Care and Hospital scope:

Hospital General Universitario Gregorio Marañón, Madrid
Hospital Universitario de Getafe, Madrid

Hospital Universitario San Cecilio, FIBAO, Granada

Hospital General Universitario de Ciudad Real, Ciudad real

Centro Fórum, Consorci Mar Parc Salut de Barcelona, Barcelona

Hospital del Mar, Consorci Mar Parc Salut de Barcelona, Barcelona

Asociación Instituto Biodonostia, Guipuzcoa

Nursing and Healthcare Research Unit (Investén-isciii) Instituto de Salud Carlos III, Madrid

IDIAP Jordi Gol, Barcelona

Complejo Hospitalario Universitario de Albacete, Albacete

Fundación del Hospital Nacional de Parapléjicos, Toledo

We will stablish the CohorFES, a prospective and observational study based on real population. Patients will be recruited from the beginning of the project to the final. Patients will be followed-up during all the project time. The capacity of the centers included in the project to recruit participants is considered adequate. All the centers have specific outpatient clinic focused on frailty and the geographic distribution around Spain decreases the bias possibility. We have a data collection platform managed by the Hospital Universitario de Getafe.
Patients in a critical situation of end of live or Barthel scale <60 would not be included.

In this cohort, Frailty Trait Scale 5 ítems will be evaluated at baseline and during the follow-up for validation its use in the clinical practice as an easy measure to be implemented in Primary Care and

hospitals.

Sample size calculation:
The calculation of the sample size in this study is based on index data obtained from the Study of Toledo of Healthy Aging. As is expected that fragility prevalence rates as well as the progression of the fragility syndrome will be superior in the clinical population that in the general population, these estimates can be considered as conservative and they allow, therefore, an additional margin of security in reaching sufficient statistical power.
Fragile rates are estimated from subjects who have a negative FTS5 that later develop fragility, which was 0.075 (7.5%). The ratio of exposed / unexposed was 1008/196 (ratio of negatives vs. positives), that is 5.1. The minimum relative risk (RR) to be detected is estimated taking as an interest variable "incident frailty" (stage transition marker) for which OR of the different estimates range between 1.78 and 5.4 and therefore we adopt a RR conservative to detect of 2. Finally, the loss of follow-up rates is estimated at 10%.
With these data, accepting an alpha risk of 0.05 and a beta risk of 0.2 in a bilateral contrast, 215 subjects in the exposed group and 1075 in the unexposed group are needed to detect a factor of risk with a minimum RR of 2. The POISSON approach has been used for this calculation.

WP1. 3
SIDIAP (www.sidiap.org) is a database that contains the computerized pseudoanonymised clinical records in Primary Care of over 80% of the population of Catalonia. The SIDIAP database includes more than 7 million patients recorded from 2006 to the end of 2018. SIDIAP is linked with the drug use registry of the Catalan Health Service (CatSalut), the regional payer of health services to identify the drugs dispensed, and also with the registry of hospital admissions (CMBD-AH, Minimal Basic Data Set at Hospital Discharge).
We will include patients registered in SIDIAP on January 1st 2007, of age 65 or above in that date, and registered in the same Primary Care Centre for at least one year prior to the inclusion date (from January 1st 2006 or before) to ensure consistent availability of clinical data. These patients will be followed up from this index date (1/1/2007) until death, migration out of Catalonia or the final date of the study.

WP2. 1 and 2
In CohorFES and SIDIAP: The cluster partition models (k-means and hierarchical clustering) will group together individuals with similar deficits and characteristics (frailty phenotypes). Then, by using pre-established criteria (gap and silhouette), the proposed clustering solution (belonging to given clusters) will be evaluated.

WP2. 3
In a second step we will assess, in a longitudinal fashion, the appearance and accumulation of deficits during the study period and identifying the clusters subgroups with more rapid progression in the CohorFES. Results of this WP2.3 will be applied to establish and compare clusters and trajectories.

WP2. 4
All individuals of the CohorFES evaluated during the project would be invited to participate in the BioBank after informed consent was obtained (Annex 1). Serum and urine samples will be collected at baseline and 1 year after their cohort inclusion.

WP3
Frailty phenotypes and patient clusters defined in the cohorFES and SIDIAP will be correlated with health outcomes such as the use of health services (both primary and secondary care), hospital admissions, and mortality.

Variables/relevant measurements

WP1
Independent variables (exposure variables)

WP1.1 and 2

- Demographic data: Date of birth, sex, race
- Weight and Height measured in Kg and Cm
- Prevalent diagnosis
- Pharmacy use
- Barthel
- Frailty Trait Scale 5 ítems (Annex 2): 1.- walking speed test 2.- grip strength 3.- Physical Activity Scale for the Elderly (PASE) 4- Body Mass Index (BMI) 5.- progressive Romberg test.
- Loss of weight in the last year
- Center for Epidemiological Studies Depression Scale (CES-D) (Annex 3).
- SPEED-AGE
- Nutrition questionnaire (Annex 4)

WP1.3
All the deficits listed in the electronic frailty index (eFI), developed and validated in the British electronic records based on the Rockwood's frailty model will be assessed (annex 6). The resulting scale, the eFI score, contains 36 items or deficits and includes variables like disability for daily life activities, sensorial deficits, polypharmacy, several morbidities, etc.

WP 2.3
Clinical and biological markers in a subgroup of rapid progression patients (250 patients): Parameters will be recorded at baseline and 1 year after patient inclusion.

- Total body Dual-Energy X-Ray Analysis by using a DXA device (Hologic Horizon Wi, Hologic®) for the measurement of Bone Mineral Density (both overall and in the usual regions of interest) and body composition (fat mass, muscle mass).

-Biological markers (these will be evaluated at baseline and 1 year after patient inclusion):

Routine laboratory parameters

Bilirubin
Acid uric levels
Plasma albumin
Haemoglobin
GFR
25 (OH) vitamin D levels
Total lymphocyte count
High-sensitivity C-reactive protein

Specific biological parameters of frailty (apart from the routine laboratory).

1)By ELISA methods

- insulin-like growth factor 1 (IGF-I)/ insulin-like growth factor-binding protein 1 (IGFBP1)
- Fibroblastic growth factor 23 (FGF-23)
- GSH/GSSG ratio
- β2 microglobulin

2) Metabolomic pathway analysis: In order to evaluate potential biomarkers related to the fragility onset, directed metabolomics methods developed by the Research Laboratory in Applied Metabolomics of the IMIM will be applied (***Improving liquid chromatography-tandem mass spectrometry determination of polycarboxylic acids in human urine by chemical derivatization. Comparison of o-benzyl hydroxylamine and 2-picolyl amine***. J Pharm Biomed Anal 2018; ***Targeting tryptophan and tyrosine metabolism by liquid chromatography tandem mass spectrometry***. J Chromatogr A 2016).Two altered families/metabolic pathways during senescence will be evaluated: (1) Energy production including metabolites of the Krebs cycle, glycolysis and degradation of fatty acids and (2) metabolism of aromatic amino acids including the serotonin, dopamine, kinurenine and neutral chain amino acids routes long (LNAA).((International Journal of Epidemiology 2013;42:1111 -1119 doi:10.1093/ije/dyt094, SCIentIfIC Reports | (2018) 8:12148 | DOI:10.1038/s41598-018-29809-5, Essays Biochem. 2017 July 15; 61(3): 379 -388. doi:10.1042/EBC20160083., Molecules **2018**, 23, 191). Methods that will be applied for the directed determination of the selected metabolites are based on liquid chromatography coupled to tandem mass spectrometry (LC-MS / MS). Serum/urine samples will be divided into aliquots to perform the different methods. After the addition of suitable internal standards, samples will be extracted according to the family of analytes to be considered. The analytes contained in the final extract will be determined by LC-MS / MS using SRM specific acquisition methods using the appropriate configuration (column, mobile phase) for each metabolic pathway. The concentration of each analyte will be obtained by external calibration using the relationship between the analyte and the internal standard as outcome.

WP3
The outcome variables that will measure the impact of the different phenotypic profiles identified both at an individual level as well as on the health system are:

-Healthcare resource use: number of Primary Care consultations, number of prescribed drugs, and number of contacts with the Secondary Care facilities during the study period.
-Non-elective hospital admissions. Number of admissions and length of stay during the study period.
-All-cause mortality.

Limitations. The main limitation of the project is the coordination of patient recruitment among all participant centers since the data homogeneity and sample size is a key factor for the establishment of the CohorFES. We will prioritize the WP1 since is the base of the study.

Application No.:

PRINCIPAL INVESTIGATOR:

HEALTH RESEARCH PROJECT APPLICATION FORM
WORK PLAN

Development stages and research team's task distribution, including tasks assigned to the technical staff. Please state the setting/centre where the project will be carried out.

(Please only use the space provided below)

WP1.1 and 2. The first 30 months of project
CohorFES establishment with a prevision to include up to 3000 subjects from participant centers (Primary Care and Hospitals). Baseline analysis of frailty phenotypes using FTS5, Fried and Rockwood's frailty model in the CohorFES. Classification in robust, pre-frailty and frailty. Patient recruitment will be performed for all clinic researchers in respective centers (see Annex 6). Patients will be followed-up during the study.
Responsable: Xavier Nogues, Leocadio Rodriguez Mañas; Data manager: Marta Pineda Moncusi, Jose Antonio Carnicero

WP1.3 At first year of the project
Study design for SIDIAP data collection and big data managing using MySQL software.
In the Hospital del MAR-IMIM Responsable: Xavier Nogues; Data manager: Marta Pineda Moncusi

WP2. 1 and 2 From second year to end of project
We will use data-driven artificial intelligence models (unsupervised machine learning algorithms) for the profiling of clusters of individuals prone to the accumulation of different given deficits. First, we will consider socio-demographic variables (age, gender, etc.) as well as the different components of the variables included in the four frailty tools (FTS5, Fried, Rockwood and eFI scale) as characteristics of the clusters. The cluster partition models (k-means and hierarchical clustering) will group together individuals with similar deficits and characteristics (frailty phenotypes). Then, by using pre-established criteria (gap and silhouette), the proposed clustering solution (belonging to given clusters) will be evaluated. The identified and evaluated clusters using this approach will be then labelled according to their defining characteristics. For example, it may be that we identify a cluster of patients with a high prevalence of sensory deficits (hearing, vision) and a different one with a high prevalence of cardiovascular and metabolic disease.
Longitudinal process to record the appearance and accumulation of deficits during the study period, or until death or loss to follow. The period of evaluation will be each 6 months for pre-frail and frail and 12 months for robust individuals.
Latent class analysis (mixed growth models or similar) will be used for the identification of differential longitudinal trajectories. These pathways can be identified due to their differentiated acceleration in the deficits acquisition (and, therefore, frailty) or by the combination of different characteristics/deficits. These models will identify clusters of individuals with different longitudinal frailty trajectories.
In the Hospital del MAR-IMIM Responsables: Xavier Nogues, Leocadio Rodriguez Mañas; Data manager: Marta Pineda Moncusi, Research advisors: Dolores Sanchez Rodriguez

WP2. 3 Throughout the project
This exploratory project aims to analyze potential biological markers in the frailty rapid progression subgroups. The definition of clusters at high risk of rapid progression offers a unique opportunity for assessing these biomarkers in groups of significant homogeneity. The signals hereby detected will constitute the proof of concept for future research studies specifically planned for their validation and clinical calibration. Indeed, the accessibility and efficient cost are the two requirements for the markers to be carried forward in frailty research. In this point the link with the WP2.4 would be important to decide in future research the lab analysis of the more relevant biomarkers resulted from it.
In the IMIM, Responsable: Natalia Garcia Giralt; Sample and Data manager: Marta Pineda Moncusi, Nicola Kielland

WP2.4 Throughout the project
Elaboration of logistic plan to create the Biobank CohorFES . Acquisition of Kits for blood and urine collection, and sample storing.
In the IMIM, Responsable: Natalia Garcia Giralt; Sample and patient management: Lourdes Cos, Abora Rial

WP3 From second year to end of project
We will use survival models (Kaplan-Meier and Cox proportional hazard models or competing risk Fine-Gray regression) to analyze the impact of the identified baseline and longitudinal frailty phenotypes on the proposed outcomes (healthcare resource use, hospital admissions, and mortality). These models will provide relative risk estimates (Hazard Ratio or similar, and 95% Confidence Intervals) for the various outcomes according to the identified frailty phenotypes.
In the IMIM Responsables: Xavier Nogues, Leocadio Rodriguez Mañas; Data manager: Marta Pineda Moncusi, Lourdes Cos

Application No.:

PRINCIPAL INVESTIGATOR:

HEALTH RESEARCH PROJECT APPLICATION FORM
WORK PLAN
(Please only use the space provided below. You may add a maximum of 8 lines of Activity/Task)

SCHEDULE/TIMELINE

ACTIVITY / TASK	PERSON/S INVOLVED	MONTHS	J	F	M	A	M	J	J	A	S	O	N	D
WP 1.1 Creation of a prospective Spanish clinical cohort (CohorFES) -3000 patients<br>WP 1.2 Validation of Frailty Trait Scale 5	Researchers in annex 6<br>MP, JAC	1º year	✓	✓	✓	✓	✓	✓	✓	✓	✓	✓	✓	✓
		2º year	✓	✓	✓	✓	✓	✓	✓	✓	✓	✓	✓	✓
		3º year	✓	✓	✓	✓	✓	✓						

ACTIVITY / TASK	PERSON/S INVOLVED	MONTHS	J	F	M	A	M	J	J	A	S	O	N	D
WP 1.3 .- SIDIAP data collection and cluster analysis	XN, MP	1º year	✓	✓	✓	✓	✓	✓	✓	✓	✓	✓	✓	✓
		2º year	✓	✓	✓	✓	✓	✓	✓	✓	✓	✓	✓	✓
		3º year												

ACTIVITY / TASK	PERSON/S INVOLVED	MONTHS	J	F	M	A	M	J	J	A	S	O	N	D
WP 2.1 describe frailty phenotypes characterized by the clustering and WP 2.2 identify longitudinal frailty pathways	MP,XN, LRM, DSR	1º year												
		2º year	✓	✓	✓	✓	✓	✓	✓	✓	✓	✓	✓	✓
		3º year	✓	✓	✓	✓	✓	✓	✓	✓	✓	✓	✓	✓

ACTIVITY / TASK	PERSON/S INVOLVED	MONTHS	J	F	M	A	M	J	J	A	S	O	N	D
WP 2.3 sample collection and assessment of clinical and biological markers that characterize rapid deterioration	NGG, MP, NK	1º year	✓	✓	✓	✓	✓	✓	✓	✓	✓	✓	✓	✓
		2º year	✓	✓	✓	✓	✓	✓	✓	✓	✓	✓	✓	✓
		3º year	✓	✓	✓	✓	✓	✓	✓	✓	✓	✓	✓	✓

ACTIVITY / TASK	PERSON/S INVOLVED	MONTHS	J	F	M	A	M	J	J	A	S	O	N	D
WP 2.4 Creation of a CIBERFES Biobank: to collect and record the samples (blood and urine)	NGG, LC, AR	1º year	✓	✓	✓	✓	✓	✓	✓	✓	✓	✓	✓	✓
		2º year	✓	✓	✓	✓	✓	✓	✓	✓	✓	✓	✓	✓
		3º year	✓	✓	✓	✓	✓	✓	✓	✓	✓	✓	✓	✓

ACTIVITY / TASK	PERSON/S INVOLVED	MONTHS	J	F	M	A	M	J	J	A	S	O	N	D
WP 3 To analyze relationships between phenotypes and health outcomes	MP, LRM, XN, LC	1º year												
		2º year				✓	✓	✓	✓	✓	✓	✓	✓	✓
		3º year	✓	✓	✓	✓	✓	✓	✓	✓	✓	✓	✓	✓

ACTIVITY / TASK	PERSON/S INVOLVED	MONTHS	J	F	M	A	M	J	J	A	S	O	N	D
Result analyses and discussion; Writing manuscripts	LRM, XN, NGG, MP	1º year												
		2º year												
		3º year	✓	✓	✓	✓	✓	✓	✓	✓	✓	✓	✓	✓

Application No.:

PRINCIPAL INVESTIGATOR:

HEALTH RESEARCH PROJECT APPLICATION FORM
WORK PLAN

Please insert a figure of the schedule (optional). **Max. 1 figure in jpg format**

Application No.:

PRINCIPAL INVESTIGATOR:

HEALTH RESEARCH PROJECT APPLICATION FORM
BACKGROUND OF THE RESEARCH GROUP

1. **Background of the research group in the area of the project.**
2. **Participation in national or international Stable Research Structures (RETICS, Platforms, CIBER, etc)**

(Please only use the space provided below)

The Musculoskeletal Research Group is a translational group composed by clinical and basic researchers with strong expertize in the age-related diseases mainly in the bone field. Our lines of research have focused on three: clinical epidemiology, bone quality and genetic studies. All these approaches are focused on the improvement of health quality of elderly people. Our research group belongs to the Internal Medicine Department of the Hospital del Mar, where a very high density of frail and elderly patients is managed.
We have a long history in the study of musculoskeletal disorders related to aging at both clinical and molecular level. In the last 10 years, our group produced >200 articles, most of them in international journals. All researches of our team are involved in research projects from competitive calls mainly in FIS grants and we have several clinical projects on-going. In this regard, four consecutive FIS projects led by the IP applicant have been successfully accomplished with a total of 16 manuscripts published in international journals. These projects were focused on the study of bone-related diseases in postmenopauseal women:
1.Study for improving life quality in breast cancer women treated with aromatase inhibitors: cohort B-ABLE
2.Genetic and clinics determinant of musculoskeletal events in postmenopausal women with early breast cancer treated with aromatase inhibitors. B-ABLE cohort
3. Aromatase inhibitors for early breast cancer. clinical and genetic effect related with osteoporosis and arthralgia
4. OPG-RANK-RANKL polymorphysm and osteoporosis relation: Functional analysis of OPGy RANKL gene polymorphysm.
Moreover, in this time, our group have supervised more than 40 students including PhD, Masters, and TFGs.

Our group has belonged to the Thematic Network of Cooperative Research in aging and fragility (RETICEF) platform (2013-2017) and nowadays we belong to the CIBER on Frailty and Healthy Ageing-CIBERFES.
Our group is expert on functional studies in cultures of human osteoblasts and osteoclasts, and in genetic studies which is demonstrated in our extensive collaboration in the GEFOS/GENOMOS consortium. In addition, we are in the manager committee of COST CA18139 action of GEnomics of MusculoSkeletal traits TranslatiOnal Network.
Our group has been pioneer in the development and clinical use of bone microindentation, and is currently actively cooperating in research in projects with groups from Norway, the United Kingdom and Australia.

Application No.:	
**PRINCIPAL INVESTIGATOR:**	

HEALTH RESEARCH PROJECT APPLICATION FORM
STRATEGIC FRAMEWORK

(Please only use the space provided below)

1. **Project's capacity of approaching the objectives and priorities of the Societal Challenge of Health, Demographic Change and Well-being of the Spanish Strategy for Science, Technology and Innovation.**
2. **Relevance of the proposal for clinical-translational research.**

The strategic objective of the project is to describe phenotypic clusters that drive through the different stages of fragility and to identify the underlaying mechanisms that finally trigger the disability. Moreover, we aim to identify the biological markers involved in the frailty progress for a early identification and monitoring the rapid deterioration of some patients.

The proposal offers:

1. An early detection of individual patients at risk of progression to frailty and dependence when fulfilling one of the identified phenotype profiles.
2. Monitoring the progression over time in the degree of frailty and vulnerability in the individual patient following an identified path of risk of evolution.
3. Offer standardized, well-defined populations where interventions to prevent or reverse the progression of frailty should be targeted.
4. Provide information for a correct identification of homogeneous clusters suitable for clinical and epidemiological research.
5. Open future research on biological markers of frailty and vulnerability progression.

The overarching goal is to minimize the impact of frailty and its consequence, dependence, in the Spanish population improving the diagnostic classification, prognostic evaluation and management strategies in different phenotypes of risk.

Application No.:
**PRINCIPAL INVESTIGATOR:**

HEALTH RESEARCH PROJECT APPLICATION FORM
AVAILABLE RESOURCES

(Please only use the space provided below)

Please detail the available resources to carry out the research study.

The IMIM (Hospital del Mar Medical Research Institute), located at the Barcelona Biomedical Research Park (PRBB) is, up to 82%, made up of research groups lead by professionals from the MAR Health Park.
Our laboratory and office are housed in the IMIM which contain all devices and infrastructures necessaries for molecular and cellular research. The IMIM provides a number of community spaces such as microscopy, histology, culture, and radioactive rooms. Moreover, there are a number of walk-in cold rooms and cryopreservation facilities.
The PRBB facilities consist of seven floors of multi research laboratories with well-equipped space including genomic, proteomic and metabolomic services, and FACS.
The musculoskeletal research unit has also a total body Dual-Energy X-Ray DXA device (Horizon Wi model HOLOGIC ® for measuring usual region of ierest and body composition, fat mass and muscle mass.
We are part of the vibrant research community that provides him with many formal and informal opportunities for scientific exchange.

Application No.:

PRINCIPAL INVESTIGATOR:

HEALTH RESEARCH PROJECT APPLICATION FORM
DETAILED BUDGET JUSTIFICATION

(Please only use the space provided below)

-Technician: The firsts 2 years of the project at morning (part time) for sample processing and preserving

-Hand Dynamometer JAMAR ®: for grip strength testing. 12 centers recruiting patients. Some centers have 2 clinic consultancies= 20, plus 10 extra for provision of replacement= 30 Units; 300 euros/unit= 9.000 euros

-SPEED-AGE walking test: We will use the walkway system (Video) which is composed of a measuring cabinet with a computer board and an informative screen. Two proximity sensors connected to the board record the start and end of the walk. A specific computer program on the board deals with the signals from the sensors, and calculates the speed. The system was developed by the Prof. Pedro Abizanda Soler's group from the Complejo Hospitalario Universitario de Albacete The system is protected under the Spanish law By the Spanish Patents and Brands Office of the Spanish Ministry of Energetics, Tourism and Digital Agenda ("Oficina Española de Patentes y Marcas, O.A., Ministerio de Energía, Turismo y Agenda Digital") in the form of "utility model" ES 1216010 and U 201830673 (year CXXXII, Number 5284, Volume II, 22 October 2018, pages 30-31). The system is quoted in 1000 euros. We plan to install one system in each Spain region (province). 1000 euros X 8 provinces= 8000 euros

-Consumable material for sample collection and storage: Prevision of 3000 patients in CohorFES. 3 euros/patient= 9.000 euros

-Sample shipping to IMIM from centers: 20 centers collecting samples x 300 euros/center= 6000 euros

-Biomarker analysis in 500 samples (250 rapid progression patients: biomarkers will be evaluated at basal and 1 year of follow-up): ELISA kits for FGF-23, β2 microglobulin, GSH/GSSG ratio, IGF-1 and IGFBP1; 10 kitsx500 euros/kit=5000 euros x 5 biomarkers= 25000 euros

-Metabolomic analysis in 500 samples: Analysis of 2 metabolic pathways in the Research Laboratory in Applied Metabolomics in the IMIM; Internal charge with public prices approved by the board of the institution (https://www.imim.cat/media/upload//arxius/tarifes/Tarif2019_lab_farmaco.pdf?_t=1549622641).
1.- Metabolism of aromatic amino acids including the serotonin, dopamine, kynurenine and neutral long chain amino acids (LNAA) routes. Price: € 50 / sample
2.- Energy production including metabolites of the Krebs cycle, glycolysis and degradation of fatty acids. Price € 41 / sample
In total: 500 samples * 91 € / sample = 45500 €

-Data retrieval, and generation of the database for the SIDIAP study: A SIDIAP project is quoted with 20.000 euros

-Publication expenses: One open acces is usually 1500 euros. Our prevision is to publish at least 3 manuscripts in these journals. 1500x3=4500 euros

-Labtop: the group will need a labtop for meetings and presentations with the other centers and for data managing 700 euros

-Congress registrations: We aim to attend 4 international congresses. Registration fees are 600-700 euros
Total 2800 euros. And 2 national congress: 500 eurosx2= 1000 euros

Application No.:	PRINCIPAL INVESTIGATOR:

HEALTH RESEARCH PROJECT APPLICATION FORM
BUDGET

Requested budget

1. Personnel costs

Personnel costs attributable to this grant will be adjusted to the salary fees established by ISCIII.

Technician 20h/week during 2 years	20.500
**Sub-total personnel costs:**	**20.500**

2. Execution costs

A) Goods and services (Equipment, consumables and other expenses)

Hand Dynamometer JAMAR ® (20 Units)	9.000
SPEED-AGE walking test (8 provinces)	8.000
Consumable material for sample collection and storage	9.000
Sample shipping	6.000
Metabolomic analysis in 500 samples (will be performed in the IMIM-internal service)	45.500
Data retrieval, and generation of the database for the study SIDIAP	20.000
ELISA kits for biomarkers assessment (500 samples)	25.000
Labtop	700
Congress registrations	3.800
Publication expenses	4.500
**Sub-total goods and services costs:**	**131.500**

B) Travel Allowance

Congress assistance (flights+hotel+diets)	5.000
**Sub-total travel and allowance costs:**	**5.000**
**Sub-total execution costs:**	**136.500**
**Total Budget Requested:**	**157.000**
**Total + 21% Costes Indirectos:**	**189.970**

Application No.:

PRINCIPAL INVESTIGATOR:

HEALTH RESEARCH PROJECT APPLICATION FORM
ANNEXES

ANNEXES (TEXT) Max. 3 pages (15,700 characters)

Annex 1

HOJA DE CONSENTIMIENTO INFORMADO

Título: Análisis de agrupaciones fenotípicas, trayectorias de progresión y factores de fragilidad y dependencia: estudio de una cohorte española CohorFES y la base de datos SIDIAP.

Financiador: *Instituto de Salud Carlos III* (ISCIII)

Investigador Principal: Xavier Nogués Solan

Números de teléfono y mail asociados:

Xavier Nogués: 932483147

IMIM: 933160497 e-mail:ngarcia@imim.es

Otros:

Esta hoja de Consentimiento Informado puede contener palabras que usted no entienda. Por favor pregunte al investigador principal o a cualquier persona del estudio para que le explique cualquier palabra o información que usted no entienda claramente.

INTRODUCCIÓN

Usted ha sido invitado a participar en un estudio de investigación. Antes de que usted decida participar en el estudio por favor lea este consentimiento cuidadosamente. Haga todas las preguntas que usted tenga, para asegurarse de que entienda los procedimientos del estudio, incluyendo los riesgos y los beneficios.

PROPOSITO DEL ESTUDIO

La fragilidad es un problema de impacto sanitario y social en progresión paralela al envejecimiento demográfico. Por ello se ha constituido en uno de los retos del siglo XXI y en una de las prioridades europeas en investigación biomédica.

Este estudio puede ayudar a validar una herramienta de diagnóstico de la fragilidad de fácil realización tanto desde el punto de vista práctico como de consumo temporal, que permita además trazar trayectorias funcionales que permitan identificar los factores que favorecen la caída a una mayor fragilidad.

PARTICIPANTES DEL ESTUDIO

El estudio es completamente voluntario. Usted puede participar o abandonar en cualquier momento sin que ello repercuta en su trato asistencial.

Para este proyecto se reclutarán pacientes de 65 años o mayores visitados en los centros médicos adscritos al proyecto.

PROCEDIMIENTOS

Se le realizará una historia clínica completa y se le preguntarán una serie de datos que se integran en escalas validadas para conocer su estado de salud y funcionalidad.

Se le realizarán cuestionarios específicos de fragilidad, y unas pruebas de fuerza, movilidad y equilibrio que no van a ocupar más allá de 15 minutos. Se le realizará una extracción de sangre y orina en cada visita para conservarlas en un Biobanco que van a permitir analizar posteriormente parámetros biológicos con las trayectorias de fragilidad.

Su participación conlleva una visita al inicio del tratamiento y cada seis meses para reevaluar su estado de salud.

RIESGOS O INCOMODIDADES

Ninguna de estas pruebas tiene riesgo para su salud ni cambiará la actitud de su médico sobre la actuación del mismo o tratamiento que precise.

El análisis de sangre va a suponer solamente la extracción de 25 ml suplementarios de sangre. Le tendremos informada de cualquier hallazgo significativo para su salud.

BENEFICIOS

Debe quedar claro que usted no recibirá ningún beneficio económico por participar en este estudio. Su participación es una contribución para el desarrollo de la ciencia y para la implementar nuevos métodos de pronóstico para mejorar la calidad de vida de las persona mayores.

PRIVACIDAD Y CONFIDENCIALIDAD

El estudio tiene una duración prevista de 3 años y ha sido aprobado por el Comité de Ética de Investigación Clínica del Parc de Salut Mar.

Le garantizamos que en todo momento se respetarán la normas éticas dictadas por la Declaración de Hèlsinki Fortaleza, Brasil, Octubre 2013, y que se garantizará la confidencialidad de los datos de acuerdo con la Ley Orgánica 3/2018, de 5 de diciembre, de Protección de Datos Personales y garantía de los derechos digitales y Reglamento (UE) nº2016/679 del Parlamento europeo y del Consejo de 27 de abril de 2016 de Protección de Datos (RGPD).

El material biológico se guardará en los Biobancos del Instituto Hospital del Mar de Investigación Médica (MARBiobanc) durante un periodo de 20 años bajo la responsabilidad del investigador principal. El material solo será utilizado para investigar sobre fragilidad y nunca se utilizará para ningún otro propósito.

A los cuestionarios, pruebas y muestras biológicas se les asignara un código de tal forma que el personal investigador, diferente a su médico, no conocerá su identidad. El equipo general de la investigación y el personal de apoyo sólo tendrá acceso a los códigos, pero no a su identidad.

Los resultados de esta investigación pueden ser publicados en revistas científicas o ser presentados en las reuniones científicas y congresos, pero la identidad suya no será divulgada. La difusión de los resultados puede realizarse posterior a la finalización del proyecto.

DERECHO A RETIRARSE DEL ESTUDIO DE INVESTIGACIÓN

Usted puede retirarse del estudio en cualquier momento. Usted tiene derecho a solicitarnos en cualquier momento que eliminemos de los registros sus datos personales y a recibir los resultados de las exploraciones que le hagan según la ley 14/2007.

Para llevar a cabo el proyecto que le hemos expuesto, las disposiciones legales vigentes aconsejan que le pidamos su

autorización.

CONSENTIMIENTO

Título: Análisis de agrupaciones fenotípicas, trayectorias de progresión y factores de fragilidad y dependencia: estudio de una cohorte española CohorFES y la base de datos SIDIAP.

Antes de firmar este documento de autorización y en cualquier momento usted puede pedir cualquier aclaración a los médicos responsables del estudio.

El Sr./ Sra. .... ha sido informada de las finalidades del presente estudio, ha podido hacer las preguntas que ha considerado oportunas y acepta participar voluntariamente en él y acepta que los investigadores del estudio obtengan el material biológico (sangre y orina) para conservarlos en los Biobancos y realizar los estudios relacionados con el estudio.

Comprendo que puedo retirarme del estudio cuando quiera, sin dar explicaciones y ello no repercutirá en la actuación del facultativo.

Nombre del/la participante   Firma participante   Firma Investigador

Fecha:

Annex 2

Frail Trait Scale 5 items y protocolo de medición y recogida estandarizada de las variables

Variables:
1.- Velocidad de la marcha
2.- Fuerza de prensión (*grip strength*)
3.- Nivel de actividad física (PASE, see below)
4- índice de masa corporal
5.- Test de equilibrio/ test de Romberg progresivo

PROTOCOLO DE MEDICIÓN DEL FTS5

1. Velocidad de la marcha. Medida como el tiempo que tarda en recorrer 3 metros desde la posición de parado. Realizar dos veces y registrar el mejor tiempo.
2. Fuerza de prensión. Medida como la fuerza de prensión palmar en el miembro dominante empleando un dinamómetro Jamar Preston. Se realizaran tres intentos descansando un mínimo de 30 segundos entre dos intentos consecutivos. Se registrará la máxima fuerza.
3. Nivel de Actividad física. Se usará el cuestionario Physical Activity Scale for the Elderly (PASE). La puntuación se obtendrá usando el algoritmo descrito en la literatura.
4. IMC: Se medirá la altura con un estadímetro (Medizintechnik seit 1890, KaWe, Alemania) redondeando al centímetro más cercano, el peso se medirá con una bascula de precisión SECA (SECA 884 floor scale, Alemania) una vez el sujeto se haya descalzado y quitado tanto objetos como ropa pesada. Para el cálculo del IMC se empleará la siguiente fórmula.
5. Test de equilibrio (SPPB). El sujeto intentará mantener el equilibrio en tres posiciones (pies juntos, semi-tándem y tándem) durante 10 segundos en este orden.

Physical Activity Scale for the Elderly (PASE)

PASE1. En los últimos siete días, ¿Cuánto participó en las actividades en las que debía de permanecer sentado, tales como la lectura, ver la TV o jugar a las cartas?

Nunca
Raras veces (1 o 2 días)
Algunas veces (3 o 4 días)
A menudo (5 a 7 días)

PASE1a. ¿Cuáles fueron esas actividades? ANOTAR EN EL PAPEL U ORDENADOR

PASE1b. Sólo contestar si en PASE1 hace alguna actividad). De media, ¿Cuántas horas al día pasa sentado haciendo esas actividades?

Menos de una hora
Más de 1 hora pero menos de 2 horas
De 2 a 4 horas
Más de 4 horas

PASE2. En los últimos siete días, ¿Con que frecuencia camino fuera de su casa o de su patio por cualquier razón. Por ejemplo, por diversión, o para hacer ejercicio, para caminar hacia el trabajo, pasear al perro, etc.?

Nunca (1 o 2 días)
Raras veces (1 o 2 días)
Algunas veces (3 o 4 días)
A menudo (5 a 7 días)

PASE2a. Sólo si caminó fuera de casa en PASE2. De media, ¿Cuántas horas al día pasa caminando?

Menos de una hora
Más de 1 hora pero menos de 2 horas
De 2 a 4 horas
Más de 4 horas

PASE3. En los últimos siete días, ¿Con qué frecuencia practica deportes ligeros o actividades de recreo, tales como bolos, golf (con coche), partidas de cartas, pescar desde una lancha u otras

actividades similares?

Nunca
Raras veces (1 o 2 días)
Algunas veces (3 o 4 días)
A menudo (5 a 7 días)

PASE3a. ¿Cuáles fueron esas actividades? ANOTAR EN EL PAPEL U ORDENADOR

____

PASE3b. Sólo si en PASE3 hace alguna actividad. De media ¿Cuántas horas al día pasa haciendo esas actividades?

Menos de una hora
Más de 1 hora pero menos de 2 horas
De 2 a 4 horas
Más de 4 horas

PASE4. En los últimos siete días, ¿Con que frecuencia realiza deportes moderados o actividades de recreo tales como dobles de tenis, danza con balón, caza, golf sin coche, fútbol, u otras actividades similares?

Nunca
Raras veces (1 o 2 días)
Algunas veces (3 o 4 días)
A menudo (5 a 7 días)

PASE4a. ¿Cuáles fueron esas actividades? ANOTAR EN EL PAPEL U ORDENADOR

____

PASE4b. Sólo si en PASE4 hace alguna actividad. De media ¿Cuántas horas al día pasa haciendo esas actividades?

Menos de una hora
Más de 1 hora pero menos de 2 horas
De 2 a 4 horas
Más de 4 horas

PASE5. En los últimos siete días, ¿Con que frecuencia realiza deportes extenuantes o actividades de recreo tales como ciclismo, natación, tenis simples, aerobic, jogging, esquí u otras actividades similares?

Nunca
Raras veces (1 o 2 días)
Algunas veces (3 o 4 días)
A menudo (5 a 7 días)

PASE6a. ¿Cuáles fueron estas actividades? ANOTAR EN HOJA U ORDENADOR

____

PASE6b. Sólo si realiza deportes moderados. De media, ¿Cuántas horas al día pasa realizando esas actividades?

Menos de una hora
Más de 1 hora pero menos de 2 horas
De 2 a 4 horas
Más de 4 horas

PASE6. En los últimos siete días, ¿Con que frecuencia realiza algún ejercicio dirigido específicamente a mejorar su fuerza muscular y resistencia, tal como levantamiento de peso o maquinas de fuerza, levantar cosas, etc.?

Nunca
Raras veces (1 o 2 días)
Algunas veces (3 o 4 días)
A menudo (5 a 7 días)

PASE6a. ¿Cuáles fueron estas actividades? ANOTAR EN HOJA U ORDENADOR

PASE6b. Sólo si realiza deportes moderados. De media, ¿Cuántas horas al día pasa realizando esas actividades?

Menos de una hora
Más de 1 hora pero menos de 2 horas
De 2 a 4 horas
Más de 4 horas

PASE7. En los últimos siete días, ¿Ha hecho algún trabajo doméstico ligero, como quitar el polvo o fregar los platos?

1. Sí 2. No 3. NS 4. NC

PASE8. En los últimos siete días, ¿Ha hecho algún trabajo doméstico pesado o quehaceres tales como pasar el aspirador, fregar suelos, limpiar ventanas o transportar leña?

1. Sí 2. No 3. NS 4. NC

PASE9. En los últimos siete días, ¿Ha realizado reparaciones domésticas como pintar, empapelar, reparaciones eléctricas, etc.?

1. Sí 2. No 3. NS 4. NC

PASE10. En los últimos siete días, ¿Ha realizado usted actividades de mantenimiento del césped o cuidado del patio o del terreno, incluido la retirada de nieve o de las hojas, cortar leña, etc.?

1. Sí 2. No 3. NS 4. NC

PASE11. En los últimos siete días, ¿Ha realizado usted actividades de jardinería exterior?

1. Sí 2. No 3. NS 4. NC

PASE12. En los últimos siete días, ¿Ha cuidado usted de otra persona, tal como un niño, cónyuge dependiente o de otro adulto?

1. Sí 2. No 3. NS 4. NC

PASE13. Durante los pasados siete días ¿Trabajó por dinero o como voluntario?

1. Sí 2. No 3. NS 4. NC

Si contesta que Si en PASE13:
PASE13a. ¿Cuántas horas por semana trabaja por dinero y/o como voluntario? _ _ _ _

PASE13b. ¿Cuál de las siguientes categorías describe mejor la cantidad de actividad física que se necesita para su profesión y/o trabajo voluntario?

1. Principalmente sentado, con ligeros movimientos de los brazos (por ejemplo, trabajo de oficina, conducir un autobús, relojero, trabajar sentado en una cadena)
2. Sentado o de pie, con algún paseo (por ejemplo, cajero, trabajo en una oficina general, trabajo con maquinaria o con instrumentos ligeros)
3. Caminando, con manejo de algunos materiales que generalmente pesan menos de 22 Kg. (por ejemplo, cartero, camarero/a, empleado de construcción, trabajos con maquinaria o instrumentos pesados)
4. Caminando y trabajo manual pesado que a menudo requiere manejar materiales que pesan más de 22 Kg. (por ejemplo, madero, albañil con piedra, granja)

Annex 3

Escala CES-D (Center for Epidemiological Studies Depression Scale)
¿Sentía que todo lo que hacía suponía un esfuerzo en la última semana?"
o
"¿Sentía que no podía ponerse en marcha la última semana?".
Pérdida de peso no intencionada en el último año mayor de 4,5 kg o mayor del 5% del peso previo en el último año.

Annex 5
List of 36 deficits contained in the Electronic Frailty Index (eFI).Clegg A, et al. Age Ageing. 2016

Activity limitation, Memory and cognitive problems, Anaemia and haematinic deficiency, Mobility and transfer problems, Arthritis, Osteoporosis, Atrial fibrillation, Parkinsonism and tremor, Cerebrovascular disease, Peptic ulcer, Chronic kidney disease, Peripheral vascular disease, Diabetes, Polypharmacy
Dizziness, Requirement for care, Dyspnoea , Respiratory disease, Falls, Skin ulcer, Foot problems, Sleep disturbance, Fragility fracture, Social vulnerability, Hearing impairment,Thyroid disease, Heart failure
Urinary incontinence, Heart valve disease, Urinary system disease, Housebound,Visual impairment
Hypertension, Weight loss and anorexia, Hypotension/syncope, Ischaemic heart disease

Annex 6

Carmen Osuna del Pozo, Hospital General Universitario Gregorio Marañón

Leocadio Rodriguez Mañas, Hospital Universitario de Getafe

Pedro López-Doriga Bonnardeaux, Hospital Universitario de Getafe

Almudena Areosa, Hospital Universitario de Getafe

Myriam Valdés, Hospital Universitario de Getafe

Jose Antonio Carnicero, Hospital Universitario de Getafe

Verónica Ávila Rubio, Hospital Universitario San Cecilio

Mónica Machón, Asociación Instituto Biodonostia

Carolina Guell, Asociación Instituto Biodonostia

Javier Iruzubieta Barragan, Nursing and Healthcare Research Unit (Investén-isciii), Instituto de Salud Carlos III

María Ángeles Caballero Mora, Hospital General Universitario de Ciudad Real

Cristina Carbonell, ABS vía roma, IDIAP Jordi Gol
Mª Dolores Sánchez Rodríguez, Centro Forum, Consorci Mar Parc Salut de Barcelona
Mª Lourdes Cos, Hospital del Mar, Consorci Mar Parc Salut de Barcelona
Albora Rial, Hospital del Mar, Consorci Mar Parc Salut de Barcelona
Xavier Nogues, Hospital del Mar, Consorci Mar Parc Salut de Barcelona
Pedro Manuel Sánchez Jurado, Complejo Hospitalario Universitario de Albacete
Teresa Flores Ruano, Complejo Hospitalario Universitario de Albacete

Application No.:

PRINCIPAL INVESTIGATOR:

ANNEXES (FIGURES) Max. 1 figure in jpg format

Annex 4. Questionnaire of adherence to the Mediterranean diet

CUESTIONARIO DE ADHERENCIA A LA DIETA MEDITERRÁNEA

Nº	Pregunta	Modo de valoración	Puntos
1	¿Usa usted el aceite de oliva como principal grasa para cocinar?	Sí = 1 punto	
2	¿Cuanto aceite de oliva consume en total al día (incluyendo el usado para freir, comidas fuera de casa, ensaladas, etc.)?	4 o más cucharadas = 1 punto	
3	¿Cuantas raciones de verdura u hortalizas consume al día? (las guarniciones o acompañamientos = 1/2 ración) 1 ración = 200g.	2 o más (al menos una de ellas en ensalada o crudas) = 1 punto	
4	¿Cuantas piezas de fruta (incluyendo zumo natural) consume al día?	3 o más al día = 1 punto	
5	¿Cuantas raciones de carnes rojas, hamburguesas, salchichas o embutidos consume al día? (ración: 100 - 150 g)	menos de 1 al día = 1 punto	
6	¿Cuantas raciones de mantequilla, margarina o nata consume al día? (porción individual: 12 g)	menos de 1 al día = 1 punto	
7	¿Cuantas bebidas carbonatadas y/o azucaradas (refrescos, colas, tónicas, bitter) consume al día?	menos de 1 al día = 1 punto	
8	¿Bebe usted vino? ¿Cuánto consume a la semana?	7 o más vasos a la semana = 1 punto	
9	¿Cuantas raciones de legumbres consume a la semana? (1 plato o ración de 150 g)	3 o más a la semana = 1 punto	
10	¿Cuantas raciones de pescado-mariscos consume a la semana? (1 plato, pieza o ración: 100 - 150 de pescado o 4-5 piezas o 200 g de marisco)	3 o más a la semana = 1 punto	
11	¿Cuantas veces consume reposteria comercial (no casera) como galletas, flanes, dulce o pasteles a la semana?	menos de 2 a la semana = 1 punto	
12	¿Cuantas veces consume frutos secos a la semana? (ración 30 g)	3 o más a la semana = 1 punto	
13	¿Consume usted preferentemente carne de pollo, pavo o conejo en vez de ternera, cerdo, hamburguesas o salchichas? (carne de pollo: 1 pieza o ración de 100 - 150 g)	Sí = 1 punto	
14	¿Cuantas veces a la semana consume los vegetales cocinados, la pasta, arroz u otros platos aderezados con salsa de tomate, ajo, cebolla o puerro elaborada a fuego lento con aceite de oliva (sofrito)?	2 o más a la semana = 1 punto	

Extraído de: Schroder et al., A short screener is valid for assessing Mediterranean diet adherence among older Spanish men and women. J Nutr 2011;141:1140–5.
